# Correlation between Prognostic Factors and the Histopathological Response to Neoadjuvant Chemotherapy in Osteosarcoma: A Retrospective Study

**DOI:** 10.1155/2021/8843325

**Published:** 2021-04-26

**Authors:** Yogi Prabowo, Iwan Setiawan, Achmad Fauzi Kamal, Evelina Kodrat, Muhammad Luqman Labib Zufar

**Affiliations:** ^1^Department of Orthopedic and Traumatology, Cipto Mangunkusumo General Hospital, Faculty of Medicine Universitas Indonesia, Jakarta, Indonesia; ^2^Department of Anatomical Pathology Cipto Mangunkusumo General Hospital, Faculty of Medicine Universitas Indonesia, Jakarta, Indonesia

## Abstract

**Background:**

Multimodality treatment, incorporating neoadjuvant chemotherapy and adjuvant chemotherapy, is the standard management plan for osteosarcoma that increases the overall survival (OS) rate. However, data regarding prognostic factors affecting the histopathological response following neoadjuvant chemotherapy is limited. *Patients and Methods.* We retrospectively reviewed patients diagnosed with osteosarcoma in our center between 2008 and 2018. We classified patient characteristics according to gender, age, tumor size, site and stage at diagnosis, site of metastasis, type of surgery, necrosis rate based on the Huvos grading system, and the number of neoadjuvant chemotherapy cycles. We divided response to neoadjuvant chemotherapy into poor responder for patients with Huvos grades 1 and 2 and good responder for patients with Huvos grades 3 and 4. We also documented patients' survival and follow-up information.

**Results:**

We reviewed 64 patients within 5–65 years of age, dominated by men (62.5%). The distal femur (53.1%) was the most common site of osteosarcoma. Fifteen (23.4%) patients had a good response while 49 (76.6%) patients were poor responders to neoadjuvant chemotherapy based on the Huvos grading system. Based on multivariate analysis, gender (*p* = 0.012), age (*p* = 0.029), symptom duration (*p* = 0.004), and tumor enlargement after neoadjuvant chemotherapy (*p* < 0.001) were significantly associated with histopathological response. A scoring system was proposed integrating these significant variables (age > 20 years = 1 point, female gender = 1 point, symptom duration > 12 weeks = 1 point, and increased tumor size after neoadjuvant chemotherapy = 2 points). This scoring system divides patients into two groups with a total score of more than two predicting a poor responder to neoadjuvant chemotherapy.

**Conclusions:**

Age, gender, symptoms duration, and tumor size after neoadjuvant chemotherapy are the prognostic features that affect the histopathological response to neoadjuvant chemotherapy in patients with osteosarcoma.

## 1. Introduction

Osteosarcoma is one of the most common orthopedic malignancies that is marked with excessive production of osteoid matrix by bone tumor cells. This condition frequently affects the metaphysis of long bones, especially at the distal femur and proximal tibia [1–5]. However, osteosarcoma is a rarely found tumor with an incidence of 4–5/1,000,000 per year [3, 6]. This neoplasm has a bimodal age distribution with the first peak and second peak in the second decade of life between 14 and 26 years and in patients over 65 years, respectively [4, 7, 8]. The growth spurt may be associated with the incidence of osteosarcoma among adolescents and young adults, while secondary malignancy becomes a common cause of osteosarcoma development in the later years. The common chief complaints or clinical symptoms of this malignancy may include locally aggravating pain and progressive swelling or lumps. Structural dysfunction and pathological fractures are commonly found in some cases.

Owing to its high invasiveness and metastatic feature, the prognosis of osteosarcoma is poor [9]. Conventional osteosarcoma with the chondroblastic, osteoblastic, fibroblastic, telangiectatic, and small-cell subtypes is more likely to develop a high-grade and highly metastatic tumor. The high-grade surface and secondary osteosarcoma subtypes may also enlarge and metastasize mainly to the lung [10, 11]. Restoring the function of the affected extremity and optimizing the patient's quality of life are considered as the main goals in addition to controlling the primary tumor and its micro-metastatic progression. Multimodality treatment which incorporates neoadjuvant chemotherapy, surgery, and postsurgery chemotherapy is indisputably required [12]. Prior to the 1970s, the treatment approach with surgery only resulted in a poor outcome with a cure rate of only <20% of patients [13]. The majority of cases led up to death. This poor outcome was mainly due to the rapid progression of osteosarcoma and lung metastasis. With the integration of multimodality treatment, the 5-year survival rate has improved to 70–80% in the last two decades [14, 15].

The neoadjuvant chemotherapy, which is integrated with the standard treatment plan nowadays, may reduce the tumor size and eliminate the micrometastases, thus prolonging event-free survival (EFS) and overall survival (OS) of the patients [16]. The neoadjuvant chemotherapy protocol consisting of doxorubicin, cisplatin, ifosfamide, and a high dose of methotrexate has been widely used and associated with an increased OS rate [17, 18]. A study from the European Osteosarcoma Intergroup (EOI) reported that, compared to the T10 modified regimen at Memorial Sloan-Kettering Cancer Center, a combination of doxorubicin and cisplatin, which was given in six cycles (18 weeks), resulted in better outcomes [19, 20].

Previous studies have assessed prognostic factors, such as gender, age, primary tumor size, anatomic location, presenting metastasis, level of serum alkaline phosphatase (ALP), level of lactate dehydrogenase (LDH), osteosarcoma types, surgical procedures, and histopathological response to neoadjuvant chemotherapy, and their correlation with patient survival rates [21–28]. The histopathological response in specific can be measured indirectly by the Huvos grading system. This grading system is based on tumor necrosis rate compared to the residual viable tumor. The Huvos grading system consists of four grades. In grade 1, there is little or no evidence of necrosis; in grade 2, there is partial response with 50–90% necrosis; in grade 3, there is 90–99% necrosis; and in grade 4, there is 100% necrosis with no viable tumor. In previous large trials, poor responders with <90% necrosis were associated with an inferior 5-year survival rate compared to good responders [16, 26–28]. The response level was also reported to be the key information in the determination of further treatment in patients with osteosarcoma.

Nevertheless, data regarding prognostic factors that affect the histopathological response following neoadjuvant chemotherapy is still limited. Therefore, in this retrospective study, we reviewed and assessed data on clinical features and laboratory parameters, including patient gender, age, tumor size, tumor site, tumor stage, metastasis site, type of surgery, percentage necrosis according to the Huvos grading system, and the number of neoadjuvant chemotherapy cycles, to identify and determine whether these factors have a significant association and prognostic significance with the histopathological response following neoadjuvant chemotherapy based on the Huvos grading system.

## 2. Methods

### 2.1. Study Design

This was a retrospective study involving patients with osteosarcoma who underwent neoadjuvant chemotherapy. We included patients diagnosed with osteosarcoma at our center between January 2008 and December 2018. The independent variables were gender, age, tumor size, tumor site, tumor stage, the presence of metastasis, the presence of pathological fracture type of surgery, ALP serum level, LDH serum level, and the number of neoadjuvant chemotherapy cycles. The dependent variable was histopathological response following neoadjuvant chemotherapy based on the Huvos grading system. We analyzed the association and prognostic significance of these independent variables in relation to the histopathological response following neoadjuvant chemotherapy.

### 2.2. Patient Population

Inclusion criteria for the study population were as follows: (1) osteosarcoma was diagnosed histologically by biopsy; (2) patients underwent neoadjuvant chemotherapy before the surgical procedure; (3) limb salvage or ablation procedure was performed following neoadjuvant chemotherapy. Nevertheless, we excluded patients with the presence of low-grade malignancy tumors, patients who died during the neoadjuvant chemotherapy period or before undergoing surgery, patients who had previously received any other form of chemotherapy regimen, and patients who did not have histopathological slide specimen for Huvos analysis. The ethics committee of our center provided ethical approval. Moreover, the guardians or the patients themselves signed a consent form regarding the use of their medical records for this study.

### 2.3. Staging and Treatment Details

The combination of various radiological studies, involving a plain film, bone scan, and magnetic resonance imaging of the affected limb, was performed for osteosarcoma staging. Moreover, metastasis extension workups including chest x-ray and computed tomography of thorax, abdomen, and pelvis region were also performed to identify invasion to other organs. Biochemical and blood analyses were obtained at the time of diagnosis and after treatment. Fine-needle aspiration biopsy, core needle, or open biopsy was performed to establish the diagnosis based on histopathological examination. The neoadjuvant chemotherapy protocol in our center consists of doxorubicin 75 mg/m^2^ and cisplatin 100 mg/m^2^. This combination was given in 1–3 cycles within 6–18 weeks, depending on the patient's condition.

The type of surgery (limb salvage or limb ablation procedure) was determined based specifically on the clinical condition, histopathological response, patient's performance status, and patient's requirements and desires. Limb salvage surgery was preferred when there was a good chemotherapy response or chemosensitive tumor, achievable good surgical margin for extensive excision, no involvement of the primary blood vessels and nerve, good performance status with a Karnofsky score of more than 60, good coverage of soft tissue areas, and patients' strong desire to retain their limbs and their functions. Close surveillance consisted of CXR examination every 3 months during the first 2 years and every 4–6 months in the following 3 years and CT thorax every 6 months.

### 2.4. Data Collection

From the from medical records, we also documented demographic and clinical data, including patient's gender, age, tumor size, tumor site, tumor stage, symptoms duration, metastasis site, type of surgery, the presence of metastasis, the presence of a pathological fracture, ALP serum level, LDH serum level, necrosis rate based on the Huvos grading system, and the number of neoadjuvant chemotherapy cycles, as well as relapse site, relapse time, survival, and follow-up information. We collected the histopathological examination assessing necrosis rate after neoadjuvant chemotherapy treatment from the report issued by the pathological anatomy department in our center. We further divided patients based on Huvos response into poor responder for patients with Huvos grades 1 and 2 and good responder for patients with Huvos grades 3 and 4.

To minimize the potential sources of bias, we already clearly identified the inclusion and exclusion criteria for the study population. Moreover, the authors who selected and analyzed the data of the patients were blinded to the outcome of osteosarcoma status. We used the standard protocols and instruments in our center to measure the variables involved in this study to prevent information bias. In addition, data from hospital medical records were also validated by using records of direct measurements data in our orthopedic and traumatology department. Furthermore, an estimated 96 patients were required for the study size based on the formula for two independent proportions for unpaired category of analytical test with type I error (*α*) = 5%, *Z*_*α*_ = 1.96, type II error = 20%, *Z*_*β*_ = 0.84, and *P*_2_ = 0.45.

### 2.5. Statistical Analysis

Correlation analysis using the Pearson chi-square test and the Fisher exact test was performed to assess the correlation between clinical and laboratory features with the histopathological response to neoadjuvant chemotherapy based on the Huvos grading system. Clinical and laboratory data were further analyzed using a backward logistic regression to determine which features are significant when calculated concurrently. The variables included in this test had a *p*-value < 0.25 in the previous bivariate correlation test. A proposed scoring system to predict response to the neoadjuvant chemotherapy was evaluated and analyzed using the Hosmer-Lemeshow test and receiver operating characteristic (ROC) curve.

## 3. Results

### 3.1. Patient Demographics and Clinical Characteristics

From 163 patients who fitted the inclusion criteria, only 64 patients with a slide specimen of histopathological response to neoadjuvant chemotherapy were available and further analyzed. The patients in this study population consisted of 40 (62.5%) men and 24 (37.5%) women. The proportion of the age of patients in the first, second, third, and fourth or more decade of life was 8 (12.5%), 40 (62.5%), 6 (9.4%), and 10 (15.6%) patients, respectively. The mean age was 19 years (range: 5–65 years). Forty-seven (73.4%) patients presented with symptoms and complaints that had been experienced for more than 12 weeks. The average duration between the onset of symptoms to the first visit to our center was 23 weeks (range: 4–96 weeks).

The proportions of tumor locations were 34 (53.1%) at the distal femur, 13 (20.3%) at the proximal of tibia, and 17 (26.6%) in other locations (distal tibia in six patients, proximal humerus in four patients, iliac wing in two patients, and distal humerus, distal radius, proximal femur, clavicle, and proximal fibula in one patient each). The osteosarcoma types found in our patients were the high-grade tumor of conventional type (stage IIB in 70.3% and stage III in 29.7% of cases) and central type osteosarcoma in 61 (95.31%) patients. Telangiectatic and small-cell osteosarcoma subtypes were found in the other three patients. Moreover, 17 (89.5%) metastasis cases were found in the lung. Fifty (78.1%) patients presented with a tumor size of more than 10 cm in diameter. Tumor enlargement, increased levels of serum ALP, and LDH after neoadjuvant chemotherapy were found in 51 (79.8%) patients, 20 (31.3%) patients, and 33 (51.6%) patients, respectively. In the present study, only 15 (23.4%) patients progressed to a good response according to the Huvos grading after neoadjuvant chemotherapy.  Table 1 shows the demographic and clinical data of the patients involved in this study.

### 3.2. Prognostic Value of Clinical Features and Laboratory Parameters in relation to Histopathological Response following Neoadjuvant Chemotherapy

The symptoms and complaints duration (*p* = 0.016), tumor enlargement following neoadjuvant chemotherapy (*p* < 0.001), metastasis, and tumor staging at diagnosis (*p* = 0.028) were proven to be significantly associated with histopathological response following neoadjuvant chemotherapy based on Pearson chi-square and Fisher exact test as shown in Table 2. Further multivariate evaluation using logistic regression analysis with backward elimination resulted in a significant correlation between gender (*p* = 0.012), age (*p* = 0.029), symptoms duration (*p* = 0.004), and tumor enlargement following neoadjuvant chemotherapy (*p* < 0.001) with histopathological response as shown in Table 3. There was no significant association with regard to the laboratory data analysis, which involved the level of preneoadjuvant chemotherapy serum ALP (*p* = 0.578), preneoadjuvant chemotherapy serum LDH (*p* = 1.000), postneoadjuvant chemotherapy serum ALP (0.703), and postneoadjuvant chemotherapy serum LDH (*p* = 1.000).

### 3.3. A Scoring System as a Prediction of Histopathological Response following Neoadjuvant Chemotherapy

**Figure 1:**
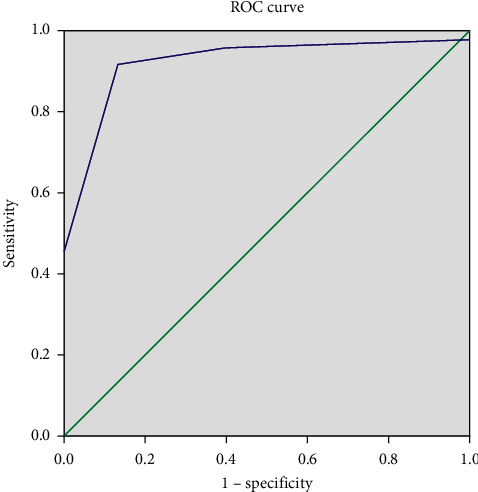
ROC curve. ROC curve in Section 3.3 showing a high value of sensitivity and specificity with larger area under the curve, and a steeper and sharper slope of the curve.

A statistical analysis using Hosmer–Lemeshow and ROC curve was performed to calibrate the proposed scoring system using the values of B and SE of each variable and the constant available in logistic regression. A total score of 2.5 was obtained and set to be the cut-off point to distinguish between the good and poor responder following neoadjuvant chemotherapy.  Table 4 shows the scoring system we proposed in the present study. Hosmer-Lemeshow showed a good calibration result with *p* = 0.063.  Figure 1 shows that a larger area under the curve and a steeper and sharper slope of the ROC curve were associated with a higher sensitivity, higher specificity, and excellent discrimination of the scoring system's cut-off point.

## 4. Discussion

Osteosarcoma is one of the most common malignant tumors in the orthopedic field. It is invasive, highly metastatic, and associated with poor prognosis. At present, the use of neoadjuvant chemotherapy before surgery followed by adjuvant chemotherapy has become the standard protocol of treatment to produce optimal outcomes. This can be related to the elimination of micrometastases and reduction in tumor size to further clarify the resection border and increase the effectivity of the surgery. Several studies have been conducted to assess and analyze factors that predict EFS and OS rates in patients with osteosarcoma. However, studies on prognostic factors affecting the histopathological response following neoadjuvant chemotherapy are limited.

To our knowledge, this is the first study to assess and evaluate the direct correlation of clinical parameters, both before and after neoadjuvant chemotherapy, with histopathological response following neoadjuvant chemotherapy. The relevance of the present study arises from the significant implication on decreased patient survival rates that a poor chemotherapy response has. In addition, this level of response will further determine the type of selection of adjuvant chemotherapy after surgery. Our main results, based on multivariate analysis, showed that gender (*p* = 0.012), age (*p* = 0.029), symptom duration (*p* = 0.004), and postneoadjuvant chemotherapy tumor enlargement (*p* < 0.001) were significantly associated with histopathological response. Moreover, we also proposed a scoring system predicting response to neoadjuvant chemotherapy based on integrating these significant variables (age > 20 years = 1 point, female gender = 1 point, symptom duration > 12 weeks = 1 point, and increased tumor size after neoadjuvant chemotherapy = 2 points). A total score of more than 2 was associated with a higher risk of poor response to the neoadjuvant chemotherapy.

Regarding patient characteristics and demographic data in the present study, the findings are consistent with those of previous studies in which the prevalence of osteosarcoma was reported to be higher in men than in women. In addition, osteosarcoma was also mostly found in the second decade of life, with the distal femur and the proximal tibia being the most commonly affected sites [20, 29–34]. This age distribution can be associated with the presence of rapid bone growth (growth spurt) in men, especially in the second decade of life, whereas women experience it at an earlier time. Most of the patients with osteosarcoma visiting our center came with long-standing symptoms or complaints and large tumor size. In addition, patients were also diagnosed with high-grade osteosarcoma (stage IIB in 70.3% of cases) or advanced stage (stage III or with metastasis in 29.7% of cases) at the first visit. All 19 patients who presented with advanced stage disease had pulmonary metastases. Two patients also had another additional metastatic site in the vertebrae and pelvis.

This phenomenon can be attributed to the low educational level and socioeconomic status of patients in developing countries that cause a poor understanding and awareness of their condition as well as a widespread culture of seeking for treatment from traditional bone healers as the first choice of a solution when there are complaints related to the muscle and bone. The patients commonly misjudged the initial complaints of pain or lumps that had not clearly enlarged in the affected area (*n* = 64, 100% of cases) as a sprain or an ordinary swelling. Patients started to seek medical help at the time when the pain was getting worse and the lump size was increased, evolving into a more severe condition. Patients were less likely to present with a fracture or history of a fracture (*n* = 6 patients, 9.6% of cases) as the major initial complaint. Furthermore, neoadjuvant chemotherapy that is currently covered by our national program has improved treatment adherence compared to the results of the previous study conducted in 2007. Our finding of 59 (92.2%) patients in the present study undergoing chemotherapy for more than 2 cycles supports this phenomenon.

In the present study, the majority of patients with osteosarcoma (*n* = 49 patients, 76.6% of cases) were reported to be poor chemotherapy responders. This finding is consistent with that of a study by Chui et al. in which the presence of a poor chemotherapy response in high-grade osteosarcoma patients was reported in 99 (60%) of cases. Moreover, this is also comparable with the findings of previous studies conducted by Prabowo et al. in 2007 with the same chemotherapy protocols [34, 35]. In addition, another study by Bishop et al. also showed similar results in which poor histological response was found in 59.1% of cases [36]. Although the study by Bishop et al. showed a similar trend of results, the chemotherapy agents were different from the regimen used in our center [6]. Bishop et al. used the combination of methotrexate chemotherapy 8–12 g/m^2^, vincristine 1.5 mg/m^2^, bleomycin 15 mg/m^2^/day, cyclophosphamide 600 mg/m^2^/day, and dactinomycin 600 mg/m^2^/day in the first protocol, and a combination of methotrexate 12 g/m^2^, cisplatin 120 mg/m^2^, adriamycin (doxorubicin) 25 mg/m^2^/day, and ifosfamide 1.8 g/m^2^/day combination in the second protocol in their study.

Nevertheless, there was contradictory result reported by other studies in which good chemotherapy response to the EOI chemotherapy regimen could reach 50.1–55.6% [19, 20]. This distinct result could be due to the difference in patient characteristics between these studies. Moreover, the non-standard chemotherapy regimen without methotrexate used at our center may also influence the different histopathological response in the present study. Methotrexate itself is one of the most active drugs and has a different mechanism of action when compared to doxorubicin or cisplatin. Methotrexate plays an important role in inhibiting dihydrofolate reductase and thymidylate depletion. This action will have a cytotoxic effect on the inability to synthesize DNA and RNA so that cancer cells cannot proliferate. Its further effect will result in apoptosis of the cancer cells.

In the present study, based on the data analysis using logistic regression test, female gender, age over 20 years, symptoms duration ≥12 weeks, and tumor enlargement following neoadjuvant chemotherapy were significant variables correlated with poor chemotherapy response. The significance of the female gender as a predictor of poor Huvos response in the present study might be due to the non-standardization of the two genders, with 21 out of the 24 female patients having an initial tumor diameter >10 cm, and 20 female patients were in the second decade of life. Conversely, a study by Prabowo et al. showed that the male gender, tumor diameter >10 cm at diagnosis, and symptoms duration ≥12 weeks were significantly correlated with poor chemotherapy response. A study by Bacci et al. reported a complete chemotherapy response, which was found in cases of the localized tumor without metastasis. In addition, they also showed that cell type and tumor tissue, as well as serum methotrexate concentration, were the key determining factors of chemotherapy response [20, 36].

In another study, the proximal tumor was reported to have a higher association with poorer chemotherapy response, whereas the association of tumor size was not statistically significant. Patients with tumors located axially or proximally usually came with a weaker condition and a more severe tumor condition; thus chemotherapy was not done completely to further proceed with immediate limb ablation surgery. A study by Whelan et al. showed that patients who underwent surgery before the scheduled time had a worse prognosis. This phenomenon could be due to the poor chemotherapy response and the possibility of micro-metastasis which eventually worsened the situation after the surgery. Furthermore, they also reported that a good response to neoadjuvant chemotherapy, a distal location of tumors, and female gender significantly correlated with increased survival rates. In multivariate analysis, limb salvage surgery was associated with an increase in five-year survival rate compared to amputation, although the value of significance was at the borderline [21].

However, no study has analyzed the direct correlation between clinical factors related to the osteosarcoma with histopathological response following neoadjuvant chemotherapy based on the Huvos grading. Previous studies usually linked the risk factors related to osteosarcoma with the OS, EFS, and progression-free survival compared to the histopathological response. Chemotherapy response, which is assessed based on the Huvos grading system, has a prognostic value that correlates significantly with patient OS. Based on this, other factors or variables that play a role in influencing the OS of patients can also be associated with the effects on neoadjuvant chemotherapy response [20–27].

A study by Miwa et al. showed a significant correlation between the absence of metastasis with a good OS rate in addition to good histologic response to chemotherapy. A study by Chui et al. reported that tumor size was also a significant factor predicting the survival prognosis of patients with osteosarcoma. Moreover, it was also reported that the viability of the remaining tumor cells more than equal to 10%, the rate of cell mitosis more than 10 per 10 HPF, and the presence of lymphovascular invasion were the prognostic factors of poor OS and EFS [34]. The presence of metastasis, micrometastases, or a large tumor size will increase the number of cancer cells so that it will require a stronger combination and a higher dose of neoadjuvant chemotherapy. In addition to the histopathological response, other studies also reported the use of 99mTc-MIBI scintigraphy and combined radiological score in assessing the chemotherapy response. Two studies showed a significant correlation with the histopathological response and OS rate [37, 38].

Nevertheless, the use of 90% necrosis as a cut-off point of good and poor histological responses to chemotherapy needs to be rethought. Basically, the use of the 90% necrosis rate limit as a cut-off between poor responders and good responders had an impact on survival rates of patients with osteosarcoma. However, based on further studies, this relationship was insignificant given the possibility of using different types of adjuvant chemotherapy between patients [39–41]. A study by Li et al. showed that the cut-off point of response rates of tumor necrosis at 90% did not correlate with OS or EFS, whereas the distribution of histological response with necrosis rates of 70% or more had a significant correlation with better OS and EFS [42]. Another study by Harting showed a significantly lower OS level found in patients with a necrosis rate of less than 50% [43]. Changes of this cut-off point can further affect the significance of the correlation between the factors predicting histopathological response following neoadjuvant chemotherapy.

Regarding laboratory data, the significance of serum ALP level as a prognostic factor of chemotherapy response might also be considered. This can be associated with the presence of osteoblast transformation and osteoclast activation, which will further interfere with the control of cell differentiation, proliferation, and degradation. These conditions will result in high serum ALP levels [44–46]. Ren et al. conducted a meta-analysis of 21 studies and concluded that an increase in serum ALP level was associated with adverse effects on OS (hazard ratio [HR] = 1.82; 95% confidence interval [CI]: 1.61–2.06) and EFS (HR = 1.97; 95% CI: 1.61–2.42). They further explained the significant correlation between high levels of serum ALP after neoadjuvant chemotherapy with lower survival rate [47]. Another study by Chen et al. also showed a similar final conclusion (HR = 178; 95% CI: 1.52–2.07) [48].

The present study has several limitations. First, most of our subjects came with large tumor sizes; therefore, the number of patients with tumor sizes less than 10 cm was insignificant. Second, in the data we analyzed, few patients had tumors in proximal locations or metastasis; hence, the relationships with these were not significant. Third, the number of cases is relatively small since only 64 of the 163 patients met the inclusion criteria. The optimal number of samples was not achieved due to the disease rarity and the absence of complete patient data in accordance with the inclusion and exclusion criteria for the analysis. Fourth, the data evaluated in the present study was from a single institution; the characteristics of the patients and study results can be specific to our institution only. Nevertheless, the present study can serve as the background literature of a direct relationship between the factors that influence neoadjuvant chemotherapy response in patients with osteosarcoma. This becomes the novelty of our study in which previous studies were more focused on the prediction of OS or EFS in patients receiving neoadjuvant chemotherapy.

## 5. Conclusion

Histopathological changes reflecting the response to neoadjuvant chemotherapy in patients with osteosarcoma can be determined by assessing the patient's age, gender, symptoms duration, and the tumor size following neoadjuvant chemotherapy treatment. Moreover, the level of serum ALP before and after neoadjuvant chemotherapy and the type of surgery have prognostic values toward OS rates in patients with osteosarcoma. The clinical features, laboratory parameters, and histopathological features of the tumor need to be well integrated to better understand the prognosis of patients with osteosarcoma. A further multicenter study, with a larger sample size, is required to produce more robust and reliable outcomes. Moreover, the remaining viable tumor cells, the level of mitotic cells, the presence of lymphovascular invasion, fibrosis or hyalinization, and a clear tumor resection border can also be used as a basis to determine histopathological response following neoadjuvant chemotherapy. Further investigation related to the validation, predictive ability, and reproducibility of the proposed scoring system needs to be performed on a larger data set.

## Figures and Tables

**Table 1:** Patient demographics and characteristics.

Features		*n*	%
Gender	Men	40	62.5
	Women	24	37.5

Age	>20 years	16	25.0
	≤20 years	48	75.0

Length of complaint	>12 weeks	47	73.4
	≤12 weeks	17	26.6

Tumor site	Distal	42	65.6
	Proximal	22	34.4

Osteosarcoma type	Conventional	61	95.31
	Telangiectatic	1	1.56
	Small cell	2	3.13

Tumor size (Ø)	≥10 cm	50	78.1
	<10 cm	14	21.9

Pathological fracture	Present	6	9.4
	Not identified	58	90.6

Preneoadjuvant chemotherapy ALP serum level	Increased	29	45.3
	Normal	35	54.7

Preneoadjuvant chemotherapy LDH serum level	Increased	63	98.5
	Normal	1	1.5

Staging	III	19	29.7
	IIB	45	70.3

Metastasis	Present	19	29.7
	Not identified	45	70.3

Postneoadjuvant chemotherapy tumor size	Enlarged	51	79.8
	Shrunk	13	20.2

Postneoadjuvant chemotherapy ALP serum level	Increased	24	37.5
	Normal	40	62.5

Increase in postneoadjuvant chemotherapy ALP serum level	Increased	20	31.3
	Decreased	44	68.7

Postneoadjuvant chemotherapy LDH serum level	Increased	63	98.5
	Normal	1	1.5

Increase in postneoadjuvant chemotherapy LDH serum level	Increased	33	51.6
	Decreased	31	48.4

Neoadjuvant chemotherapy number of cycles	<2 cycles	5	7.8
	≥2 cycles	59	92.2

Interval of neoadjuvant therapy to surgery	>4 weeks	33	51.6
	≤4 weeks	31	48.4

Surgical procedure	Ablation	27	42.2
	Salvage	37	57.8

Huvos response	Poor	49	76.6
	Good	15	23.4

**Table 2:** Univariate analysis of clinical features and laboratory parameters with histopathological response based on Huvos grading system.

Features	Huvos response								
		Poor		Good		CI 95%			
		*n*	%	*n*	%	*p*	RR	min	Max
Sex	Male	28	70.0	12	30.0	0.110	0.800	0.621	1.030
	Female	21	87.5	3	12.5				

Age	>20 years	15	93.8	1	6.2	0.089^*∗*^	1.324	1.061	1.651
	≤20 years	34	70.8	14	29.2				

Length of complaint	>12 weeks	40	85.1	7	14.9	0.016^*∗*^	1.461	1.011	2.556
	≤12 weeks	9	52.9	8	47.1				

Tumor site	Distal	33	78.6	9	21.4	0.600	1.080	0.800	1.459
	Proximal	16	72.7	6	27.3				

Tumor size (in diameter)	≥10 cm	41	82.0	9	18.0	0.075	1.435	0.895	2.300
	<10 cm	8	57.1	6	42.9				

Postneoadjuvant chemotherapy tumor size	Enlarged	45	88.2	6	11.8	<0.001^*∗*^	2.868	1.261	6.521
	Shrunk	4	30.8	9	69.2				

Staging	III	18	94.7	1	5.3	0.028^*∗*^	1.375	1.100	1.719
	IIB	31	68.9	14	31.1				

Metastasis	Present	18	94.7	1	5.3	0.028^*∗*^	1.375	1.100	1.719
	Not identified	31	68.9	14	31.1				

Pathological fracture	Present	6	100.0	0	0.0	0.322^*∗*^	1.349	1.159	1.570
	Not identified	43	74.1	15	13.6				

Preneoadjuvant chemotherapy ALP serum level	Increased	24	82.8	5	17.2	0.578	1.073	0.840	1.371
	Normal	27	77.1	8	22.9				

Preneoadjuvant chemotherapy LDH serum level	Increased	50	79.4	13	20.6	1.000	0.794	0.700	0.900
	Normal	1	100.0	0	0.0				

Postneoadjuvant chemotherapy ALP serum level	Increased	19	79.2	5	20.8	0.703	1.056	0.804	1.386
	Normal	30	75.0	10	25.0				

Increase in postneoadjuvant chemotherapy ALP serum level	Increased	18	90.0	2	10.0	0.117^*∗*^	1.277	1.004	1.625
	Decreased	31	70.5	13	29.5				

Postneoadjuvant chemotherapy LDH serum level	Increased	48	76.2	15	23.8	1.000^*∗*^	762	0.664	0.875
	Normal	1	100.0	0	0.0				

Increase in postneoadjuvant chemotherapy LDH serum level	Increased	26	78.8	7	21.2	0.338	1.145	865	1.515
	Decreased	22	71.0	9	29.0				

Neoadjuvant chemotherapy number of cycles	<2 cycles	4	80.0	1	20.0	1.000^*∗*^	1.049	0.662	1.663
	≥2 cycles	45	76.3	14	23.7				

Interval towards surgery postneoadjuvant chemotherapy	>4 weeks	27	25.3	6	7.7	0.306	1.153	0.874	1.520
	≤4 weeks	22	71.0	9	29.0				

Surgical procedure	Ablation	21	77.8	6	22.2	0.845	1.028	0.783	1.349
	Salvage	28	75.7	9	24.3				

**Table 3:** Multivariate analysis of clinical features and laboratory parameters with histopathological response based on Huvos grading system, and value of B and SE in each variable based on logistic regression analysis test.

Features	*p*	B	SE	B/SE	(B/SE)/(the smallest value of B/SE)
Gender	0.012	2907	1402	2.07	1.11
Age	0.029	2676	1437	1.86	1.00
Length of complaint	0.004	2662	1030	2.58	1.39
Postneoadjuvant chemotherapy tumor size	<0.001	3635	1082	3.36	1.80

**Table 4:** Proposed scoring criteria for an integrated prediction of histopathological response to neoadjuvant chemotherapy.

Features		Score
Gender	Women	1
	Men	0

Age	>20 years	1
	≤20 years	0

Length of complaint	>12 weeks	1
	≤12 weeks	0

Postneoadjuvant chemotherapy tumor size	Enlarged	2
	Shrunk	0

Prognostic values		Total score
Poor responder		>2
Good responder		0–2

## Data Availability

The data used to support the findings of this study are available from the corresponding author upon request.
